# Coating the plate with antibiotic cement to treat early infection after fracture fixation with retention of the implants: a technical note

**DOI:** 10.1186/s12891-018-2285-2

**Published:** 2018-10-10

**Authors:** Xu-sheng Qiu, Bei Cheng, Yi-xin Chen, Xiao-yang Qi, Wei-ping Sha, Guo-zhao Chen

**Affiliations:** 1 Department of Orthopaedics, Nanjing University Medical School Affiliated Nanjing Drum Tower Hospital, No. 321 Zhongshan Road, Nanjing, China; 2 Department of Orthopaedics, The First People’s Hospital of Zhangjiagang, No. 68 Jiyangxi Road, Zhangjiagang, China

**Keywords:** Antibiotic impregnated bone cement, Local antibiotic therapy, Infection, Fracture

## Abstract

**Background:**

Local antibiotic therapy has gained increasing attraction in the prevention and treatment of fracture infection. However, no reports have used local antibiotic therapy in the management of early infection after fracture fixation with retention of implants.

**Methods:**

The present surgical technique report the use of antibiotic impregnated bone cement in the management of early infection after fracture fixation. Initially, the fractures were fixed with plates. The average time from initial procedure to debridement was15 days (range 9 to 25 days). The infections were treated with irrigation, debridement, and retention of the implant. The lateral surface of the plates was coated with antibiotic cement and the bone defect was filled with antibiotic cement spacer after thorough debridement.

**Results:**

Ten patients underwent this technique. The mean follow-up was 2.0 years (range 6 months to 4 years). The bone union rate was 100%, and the average time to bone healing was5.5 months.There was recurrence of infection in one patient before bone healing, but the implants were left in place until bone healed, and the infection was eradicated after implant removal.

**Conclusion:**

Coating the plate with antibiotic cement is a simple technique which may play a role in the management of early infection after fracture fixation.

## Background

Approximately two million fracture-fixation devices are implanted every year in the United States [1]. With the increase in number of surgical treatments, the absolute number of implant-related infections of these surgical interventions will also inevitably rise. Implant-related infection remains the most challenging complications in peri-operative fracture care, and may result in delayed healing, lead to permanent loss of function, or even amputation in otherwise healthy patients [2]. Furthermore, the reported socio-economic effect is significant; Darouiche et al. report that in cases of implant-related infection, the costs per patient could be as high as 15,000 USD [1].

According to the time of onset, the infection after fracture fixation (IAFF) was classified into three groups: early (less than 2 weeks), delayed (2–10 weeks), and late onset (more than 10 weeks) [3]. Two main strategies could be performed in the treatment of IAFF [4]: (1) Irrigation, debridement, and retention of the implant combined with antibiotic therapy; or (2)debridement, implant removal or exchange (one or multiple stages) with accompanied antibiotic therapy. The early onset infections are commonly treated with irrigation, debridement, and retention of the implant combined with antibiotic therapy; while the late onset infections are commonly treat with debridement and implant removal or exchange (one or multiple stages) with accompanied antibiotic therapy.

Due to the advantage of achieving very high local concentration of antibiotics with low overall systemic toxicity, local antibiotic therapy has gained increasing attention in the prevention and treatment of fracture infection. Local antibiotic therapy has been shown to reduce acute and chronic infections in the most severe fracture cases [5]; also it has been used in treating late onset infections with implant removal or exchange [6–8]. However, it is surprising that no local antibiotic therapy has been consistently used in the treatment of early onset infection with retention of the implant [8, 9]. Antibiotic-impregnated bone cement has been used in the treatment of early onset infection in our center and we report the surgical technique and the preliminary clinical outcomes in a series of patients in the present study.

## Methods

The patients with closed fractures and fixed with plates within 3 weeks of injury and developed infection within 4 weeks after fixation were potential cases to received this technique. If the patients had implants removed during debridement because the fixation was unstable, they were excluded (two patients). If some screws were loose and removed, while the fixation was still stable, the patient was included. Finally, 10 patients were included. Of these patients, 4were tibial infections (2proximal, 1 shaft, and 1 distal),2 were femoral infection (2 distal), 3 were humeral infections (2 proximal, 1 distal), and 1 a calcaneal infection. The mean age was 41 years (range 11–64 years old).The average time from injury to definitive operative fracture management was 11.5 days (range 5–21 days), and the average time from initial surgery to the second debridement procedure was 15 days (range 9–25 days). Preoperative diagnosis of infection was made according to purulent drainage from the wound or wound breakdown. The purulent drainage was sent for culture. This helped with making a definitive diagnosis as well as deciding on the appropriate antibiotic to mix with the cement at the time of surgery.

All patients were treated by the senior author (CYX).All patients gave informed consent for inclusion in the study. The study was authorized by the local ethical committee and was performed in accordance with the ethical standards of the 1964 Declaration of Helsinki.

### Surgical procedure and postoperative care

**Fig. 1:**
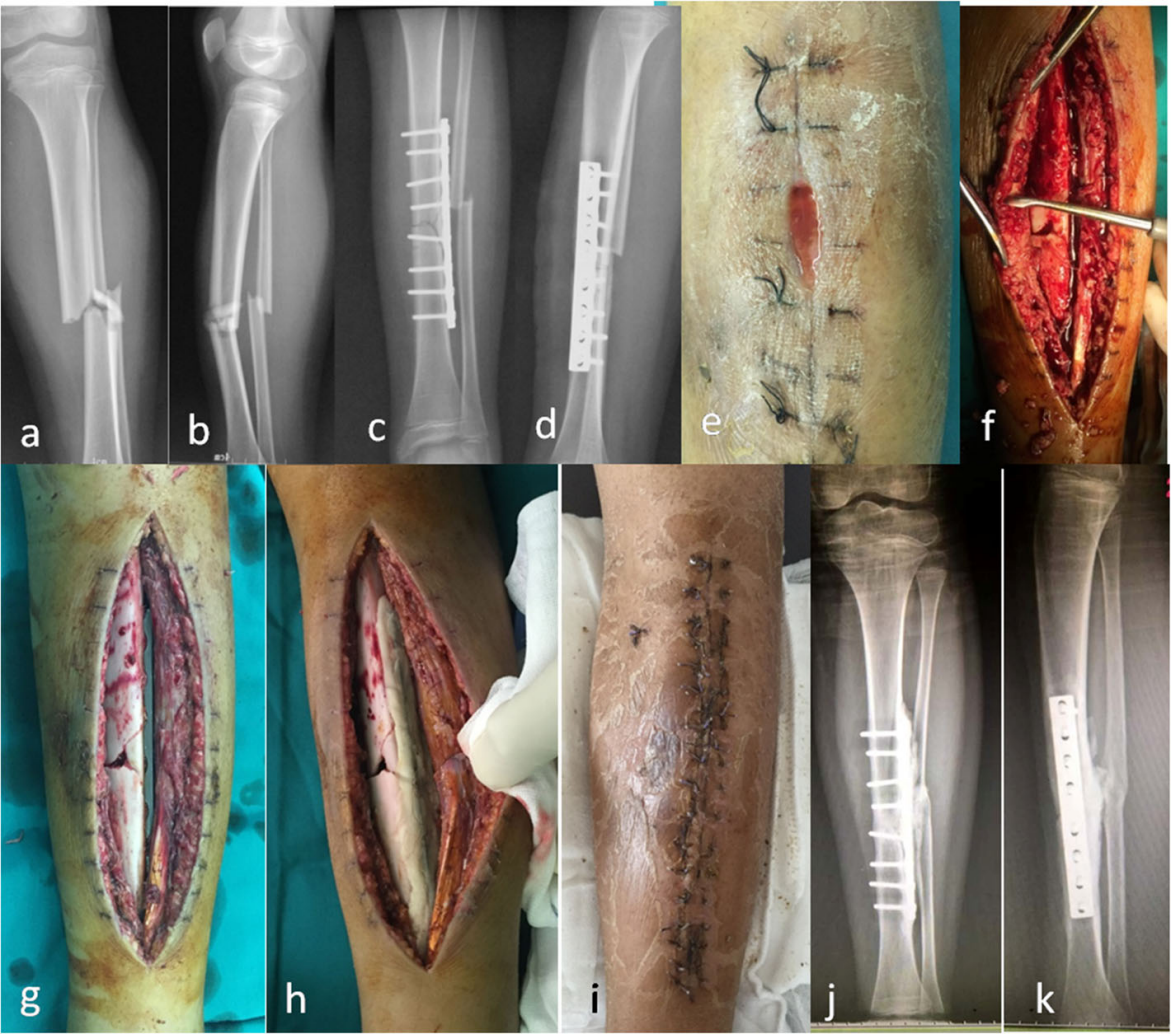
A 11-year-old male suffered an infection of the tibia after fracture fixation, he was treated with coated plate technique. The tibia fracture (**a-b**) was fixed with plate in the local hospital (**c-d**). He suffered an infection 9 days after surgery, the wound was breakdown and purulent drainage was seen (**e**). A thorough debridement was performed (**f**), the implant was still stable after debridement (**g**), therefore it was maintained and coated with antibiotic cement (**h**). The wound healed uneventfully after the surgery (**i**). The bone healed and no recurrent infection was seen at last follow-up (**j-k**)

**Fig. 2:**
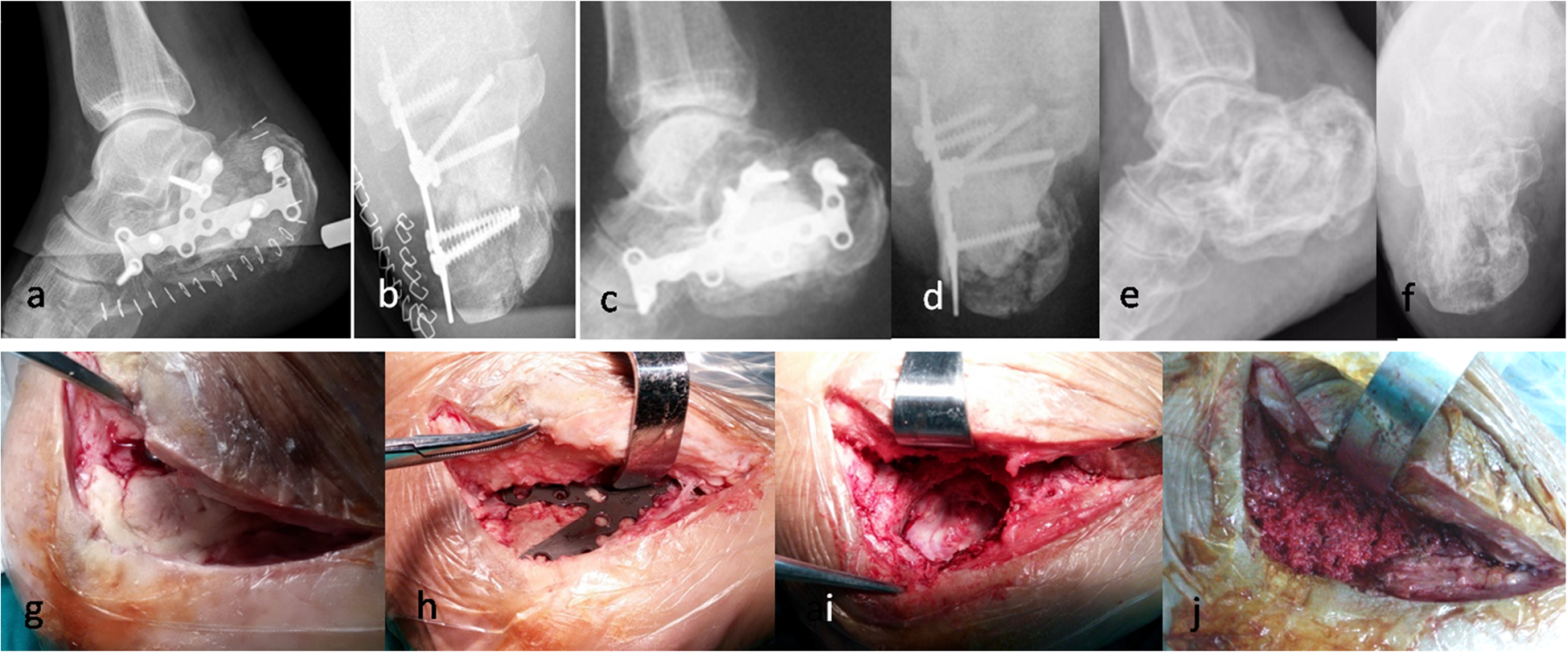
A 59-year-old male suffered calcaneus infection after fracture fixation, and was treated with irrigation, debridement, and retention of the implant combined with systemic and local antibiotic therapy. X-ray after fracture fixation showed the fracture was comminuted (**a-b**). 25 days after initial surgery, a thorough debridement was performed because of infection, two loose screws were removed, the plates were coated with antibiotic cement, and the bone defect was filled with antibiotic cement spacer (**c-d**). 8 months later, the bone cement(**g**) and the implants (**h**) were removed, and the cavity bone defect was filled with iliac bone grafts (**i-j**). At last follow- up, the bone was healed (**e-f**)

**Fig. 3:**
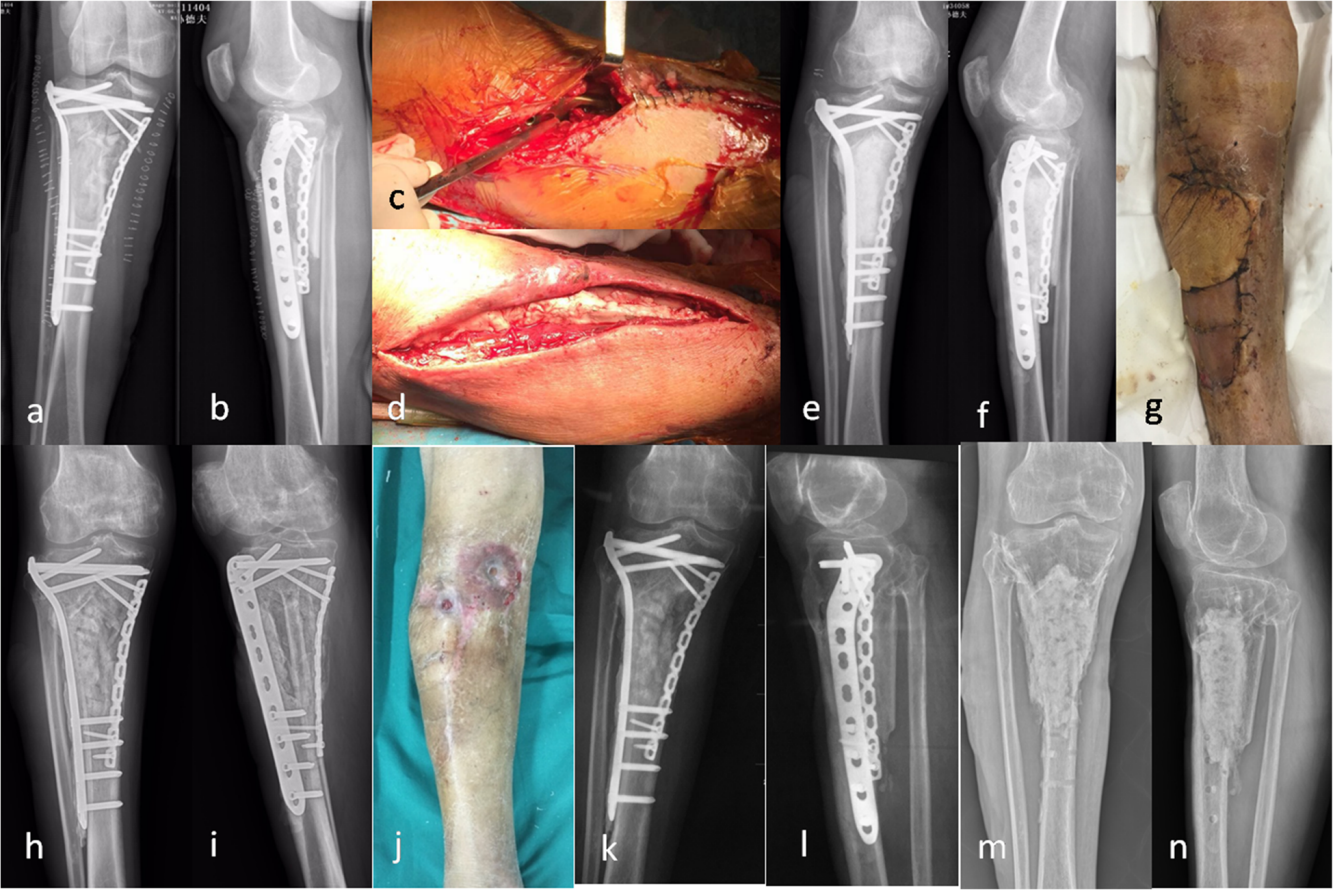
A 64-year-old male suffered an infection of the proximal tibia 15 days after fracture fixation (**a-b**). A thorough debridement was performed (**c**). The plates were coated with antibiotic cement, and the bone defect was filled with antibiotic cement spacer (**d-f**). Because of compromised soft-tissue envelope, soft tissue reconstruction was performed (**g**). Two months later, the cement was removed from the bone defect through medial approach and the bone defect was filled with iliac bone grafts (**h-i**). However, the infection recurred 6 months after bone grafting (**j**). The implants were left in place until cortical bone healed (**k-l**). Then, the implants were removed and through debridement was performed, the cavity bone defect was filled with bone cement again. No recurrent infection was seen at last follow-up (**m-n**)

Deep tissue samples were taken for microbiological analysis after exposure through the original surgical incision. Then a thorough debridement was performed, all the dead soft tissue and bone was removed (Fig. 1). If some screws were loose (always near the fracture site), they were also removed (Fig. 2). Then the lateral surface of the plates was coated with antibiotic cement (Figs. 1, 2 and 3) and the bone defect was filled with antibiotic cement spacer (Figs. 2 and 3). The coated cement was about 2–3 mm in thickness to insure that it would not interfere with wound closure. We used poly-methyl methacrylate which was premixed with gentamycin (Smith & Nephew, TN, USA) and added 2 g of vancomycin to the powder before mixing the powder and liquid. One patient needed soft tissue reconstruction, and Our plastic surgery colleagues assisted on cases requiring cutaneous/fasciocutaneous flaps for soft-tissue coverage.

Postoperatively, the patients were initially treated with intravenous antibiotics according to pre-operative culture result. Then the antibiotics were adjusted according to the sensitivities from the deep tissue culture results. If the culture was negative, the patient received vancomycin intravenously. The intravenous antibiotic treatment continued until soft tissue healing,then the patients received oral antibiotic treatment for an additional three months. All patients were evaluated postoperatively every 1–2 months.

## Results

Deep tissue cultures obtained during the surgery were positive in 9 patients, revealing the presence of *Staphylococcus aureus* (*n* = 8), *Staphylococcus epidermidis* (*n* = 1). All of these pathogens were sensitive to vancomycin.

The wounds healed uneventfully in all the patients after the surgery. The mean hospital stay was 42.4(range, 33 to 54) days.

The average follow-up was 2.0 years (range 6 months to 4 years).The bone union rate was 100%, and the average time to bone healing was 5.5 months. Two patients exhibited bony defects after debridement. The estimate volumes of bone defects were 25 cm^3^ (15–35 cm^3^). For the patients with bone defects, one patient (calcaneus) underwent removal of the implants and bone cement after bone healing, then the bone defect was filled with iliac bone grafts (Fig. 2).For another patient (proximal tibia), bone grafting was performed before bone healing and implants removal; unfortunately, the infection recurred during follow-up (Fig. 3). However, the implants were left in place until bone healed. After bone healed, the implants and bone cement were removed, the infection was eradicated.

## Discussion

The present study showed that the success rate in bone healing and eradicating infection were 100% and 90%, respectively. Berkes et al. [9] reported the bone union rate in 121 patients (123 infections) who suffered from infection within six weeks after internal fixation of acute fractures. Seventy-one percents (86 patients, 87 infections) had fracture union with operative debridement, retention of hardware, and culture-specific antibiotic treatment and suppression. Twenty-six of those 87 infections eventually underwent hardware removal after radiographic union was achieved because of recurrence of infection. Therefore, the actual infection control rate was 50.4%. Rightmire et al. [8] performed a similar study, in which 69 patients who had an acute infection within 16 weeks after definitive fixation were included. With 68% (47/69) achieving successful union. Of those patients, 28 patients required hardware removal because of persistent (18 patients) or recurrent infections (10 patients).Therefore only 27.5% of the original study group achieved healing and was free of infection after debridement, retention of hardware, and antibiotic treatment. The high success rate in eradicating infection and bone healing in the present study may due to a few reasons. First, most of the patients in the present study were early onset patients. In the early stage of infection after fracture fixation, biofilm formation appears to be in an immature stage, and obvious osteomyelitis is often not yet present [10]. Thorough debridement may eradicate most of the bacteria and/or biofilm, the residual bacteria and/or biofilm could be eliminated or suppressed by antibiotic treatment. Second, there was no open fracture, intramedullary nail, or difficult to treat pathogen in the present study, as all these factors often contribute to the treatment failure [4, 8, 9]. Third, two patients who had plates removed were excluded, they may be the most severe infection cases. Finally, the use of local antibiotic therapy may also play a role in the treatment of early infection.

Implant-related infections are typically caused by biofilm forming bacteria [11]. These infections are difficult to treat due to two reasons. First, systemic antibiotics may not reach high enough concentrations to eradicate biofilm due to local vascular damage. Second, host defenses are unable to eradicate these microorganisms, as these microorganisms are protected by a highly hydrated extracellular matrix [12]. Antibiotic-impregnated bone cement releases high local concentration of antibiotics; therefore, local antibiotic therapy could be a potential therapeutic approach in treatment of implant-related infections. Recently, a meta-analysis showed that patients with open tibia fractures would benefit from local antibiotic therapy [13]. Local antibiotic therapy has also been used in the treatment of chronic osteomyelitis [14]. In the present study, we also showed that patients with early infection after fracture fixation may also benefit from local antibiotic therapy.

In addition to the high local concentration of antibiotics released from bone cement, the heat generated during bone cement exothermic polymerization may also play an important role in eliminating bacterial biofilms. As Pihl et al. [15] demonstrated, nanoplasmonic generated heat could eliminate as high as 97% of the early biofilm bacteria after 5 min of near infrared radiation (LED emission peak at 850 nm) on the gold nanorod coated surfaces. The temperatures on bacterial suspensions with gold rods after radiation range from 48 °C to 100 °C [16, 17], which is similar to the temperatures during bone cement exothermic polymerization [18]. Therefore, it is reasonable to assume that the heat generated during bone cement exothermic polymerization may also play an important role in eliminating bacterial biofilms on implants.

The present study demonstrated that local antibiotic therapy may play an important role in the treatment of early infection after fracture fixation. However, there are many factors that influence the treatment outcome of early infection after fracture fixation, such as soft tissue condition, fracture type, implant choice, and patient’s medical comorbidities. Therefore, a randomised controlled trial is needed to confirm that the patients with early infection after fracture fixation would benefit from local antibiotic therapy.

## Conclusion

Coating the plate with antibiotic cement is a simple technique, which could be used in the treatment of early infection after fracture fixation combined with irrigation, debridement. The preliminary results in the present study was encouraging, which indicated that this technique may play a role in the management of early infection after fracture fixation.

## Abbreviations

IAFF: Infection after fracture fixation
PMMA: Polymethylmethacrylate
